# Identification of Pathways Associated with Placental Adaptation to Maternal Nutrient Restriction in Sheep

**DOI:** 10.3390/genes11091031

**Published:** 2020-09-02

**Authors:** Ashley K. Edwards, Kathrin A. Dunlap, Thomas E. Spencer, M. Carey Satterfield

**Affiliations:** 1 Department of Animal Science, Texas A&M University, College Station, TX 77843, USA; akedwards@agcenter.lsu.edu (A.K.E.); kdunlap@tamu.edu (K.A.D.); 2 Division of Animal Sciences, University of Missouri, Columbia, MI 65211, USA; spencerte@missouri.edu

**Keywords:** placentome, sheep, nutrient restriction, gene expression, immune response

## Abstract

Maternal nutrient restriction impairs placental growth and development, but available evidence suggests that adaptive mechanisms exist, in a subset of nutrient restricted (NR) ewes, that support normal fetal growth and do not result in intrauterine growth restriction (IUGR). This study utilized Affymetrix GeneChip Bovine and Ovine Genome 1.0 ST Arrays to identify novel placental genes associated with differential fetal growth rates within NR ewes. Singleton pregnancies were generated by embryo transfer and, beginning on Day 35 of pregnancy, ewes received either a 100% National Research Council (NRC) (control-fed group; *n* = 7) or 50% NRC (NR group; *n* = 24) diet until necropsy on Day 125. Fetuses from NR ewes were separated into NR non-IUGR (*n* = 6) and NR IUGR (*n* = 6) groups based on Day 125 fetal weight for microarray analysis. Of the 103 differentially expressed genes identified, 15 were upregulated and 88 were downregulated in NR non-IUGR compared to IUGR placentomes. Bioinformatics analysis revealed that upregulated gene clusters in NR non-IUGR placentomes associated with cell membranes, receptors, and signaling. Downregulated gene clusters associated with immune response, nutrient transport, and metabolism. Results illustrate that placentomal gene expression in late gestation is indicative of an altered placental immune response, which is associated with enhanced fetal growth, in a subpopulation of NR ewes.

## 1. Introduction

Maternal nutrient restriction during pregnancy impairs placental and fetal growth in humans and livestock species, often resulting in intrauterine growth restriction (IUGR) [1,2,3]. Indeed, undernutrition in ruminant livestock species is a global challenge, with the nutrient intake of ewes frequently being less than 50% of the National Research Council (NRC) recommendations [2,4]. IUGR is a leading cause of neonatal morbidity and mortality in livestock species, as well as humans, with the clinical definition of IUGR being below the 10th percentile for birthweight [1,2,5]. The intrauterine environment is not only a major determinant of fetal growth, but of the etiology of chronic disease during adult life [6,7]. In response to reduced nutrient delivery from the dam, the fetus undergoes a number of adaptations to reset critical metabolic and physiologic functions that will allow for enhanced survival in postnatal life [6,8]. The mechanisms regulating this adaptation in fetal growth, development, and programming are not fully understood.

Placental growth and development occur primarily during the first half of gestation and are significantly affected by maternal nutrition and other environmental stressors that induce epigenetic changes [9,10]. During mid-gestation, vascularization of the ruminant placenta increases markedly, especially within the cotyledonary portion of placentomes, to develop a sufficient absorptive area for nutrient exchange [10,11,12]. The functional capacity obtained during placental development is necessary to support the substantial fetal growth that occurs late in gestation. Throughout pregnancy, the placenta facilitates transport of nutrients from mother to fetus; however, nutrient delivery is dynamic and dependent upon nutrient availability, uteroplacental blood flow, placental metabolism, and transport capacity of the uterus and placenta. Not surprisingly, a significant positive correlation exists between placental and fetal weight as well as between uteroplacental blood flow and fetal weight in various species [3,13,14,15]. Interestingly, the highly adaptable placenta is hypothesized to undergo developmental and functional compensation during times of suboptimal nutrition [16].

In addition to maternal nutrient restriction resulting in smaller offspring at birth, a wider variation in lamb weights has been observed from ewes that received 50% NRC compared to those that received 100% NRC requirements. Similarly, placental weights vary greatly between uniformly treated ewes [17]. Previous work from our laboratory has shown that lamb birth weights in ewes fed at 50% of NRC requirements vary more in comparison to those receiving 100% NRC [15]. Due to this variation, lambs from ewes receiving 50% NRC requirements were further divided into the top and bottom quartiles based on fetal weights; the six largest (nutrient restricted (NR) non-IUGR) and six smallest (NR IUGR) fetuses. Results revealed decreases in placentome weight, volume, and surface area in IUGR fetuses from NR ewes compared to non-IUGR fetuses as well as lower mRNA expression of candidate nutrient transporters [15]. Available evidence suggests that adaptive mechanisms exist in a subset of nutrient restricted ewes that allows them to support normal fetal growth despite limited nutrient availability. Therefore, the objective of the present study was to utilize a discovery-based approach to identify novel placental genes associated with differential rates of fetal growth within nutrient restricted ewes.

## 2. Materials and Methods

All experimental procedures were in compliance with the Guide for the Care and Use of Agriculture Animals in Research and Teaching and approved by the Institutional Animal Care and Use Committee of Texas A&M University (AUP#2011-110).

### 2.1. Animals, Experimental Design, and Tissue Collection

Prior to embryo transfer, recipient multiparous Suffolk ewes (3–6 years of age based on dental evaluation) of similar frame size were fed 100% of their NRC requirements to maintain body condition. Ewes were synchronized into estrus using an Eazi-Breed Controlled Intravaginal Drug Releasing (CIDR) Device (Zoetis Inc., Kalamazoo, MI, USA) for 12 days. Superovulation of Suffolk donor ewes of average body condition (*n* = 14) was achieved via twice daily (0700 and 1900 h) injections of follicle stimulating hormone (FSH; Vetoquinol, Fort Worth, TX, USA) from Days 9 through 12 after CIDR insertion. Dosage decreased daily (168, 133, 105 and 70 IU, respectively) with a total dosage of 476 IU. For donor ewes, the CIDR was removed on the evening of Day 11, ewes were administered 15 mg Lutalyse (Zoetis Inc.) i.m., and, upon detection of estrus, donors were mated to 3 half-sibling Suffolk rams over a 24 h period. The CIDR was removed from recipient ewes on the morning of Day 11 and ewes were administered 20 mg Lutalyse. Recipient estrus was detected using vasectomized rams and recorded. 

Embryos were collected from donor ewes by flushing the uteri on Day 6 post-estrus as previously described [15]. Briefly, ewes were withheld from feed and water 24 h, anesthetized via i.v. anesthetic cocktail and inverted for safe insertion of the laparoscope, which was utilized to view the ovaries. A 7 cm incision was made adjacent to the midline, 5 cm below the mammary gland, and the uterus was externalized. A Foley catheter (8 Fr, 5 cc balloon) was inserted into the uterine horn, and each horn was flushed independently with 30 mL of Vigro Complete Flush medium (AB Technology, Pullman, WA, USA). Only high quality (Grade 1) morulae or blastocysts with an intact zona pellucida were used for the study. At the time of embryo transfer, recipients were withheld from food and water for 24 h, anesthetized with an i.v. anesthetic cocktail, and inverted. The ovaries were viewed laparoscopically, and Babcock forceps were utilized to grasp the uterine horn. The tip of the ipsilateral uterine horn was exteriorized, and a single embryo was transferred into the uterine lumen of each recipient ewe.

Pregnancy was diagnosed by ultrasound on Day 28 of pregnancy. On Day 35 of pregnancy, ewes were assigned randomly to a control-fed group (100% NRC; *n* = 7) and a total caloric nutrient-restricted group (50% NRC; *n* = 24). Composition of the diet has been published previously [18]. All ewes were individually housed on concrete flooring from Days 28 to 125 of pregnancy and fed once daily at 0700. Beginning on Day 28, body weight was determined every 7 days and feed intake was adjusted based on changes in body weight. 

On Day 125 (term = 147 days), ewes were necropsied and conceptus (fetal-placental unit) development assessed. At the time of necropsy ewes were euthanized using Beuthanasia (Merck Animal Health, Kenilworth, NJ, USA) administered i.v. to effect. Following euthanization, the fetus was removed, measured, and dissected to obtain organs. A portion of the uteroplacental-unit was removed and placentomes were snap frozen in liquid nitrogen. The remainder of the uteroplacental unit was filled with warmed PBS with lidocaine and maternal and fetal arteries were catheterized to allow for perfusion of placentomes with Carnoy’s solution as previously described [19]. Placentomes were dissected, counted, and weighed following perfusion. 

Fetuses from ewes fed 100% NRC formed the control group (*n* = 7). Fetuses within the NR group (*n* = 24) were segregated into quartiles based on fetal weight distribution at Day 125. The highest (NR nonIUGR; *n* = 6) and lowest (NR IUGR; *n* = 6) quartiles were selected for further investigation as described previously [15,20].

### 2.2. RNA Extraction and Affymetrix GeneChip Array Analysis

Total cellular RNA was isolated from frozen placentomes using Trizol reagent (Gibco-BRL, Bethesda, MD, USA) according to manufacturer’s instructions. Total RNA samples were digested with RNase-free DNase I and cleaned up using the RNeasy MinElute Cleanup Kit (Qiagen, Valencia, CA, USA). Quality and quantity of RNA were determined using the Agilent Bioanalyzer (Agilent Technologies, Santa Clara, CA, USA) and the NanoDrop 1000 (Thermo Fisher Scientific, Inc., Wilmington, DE, USA), respectively. Only samples with an RNA integrity number (RIN) > 8.0 were used for microarray analysis. New RNA extractions were performed for samples not meeting the RIN requirements so that all animals (*n* = 6 per group) were included in the microarray analysis.

Microarray analyses were performed on placentomes from NR non-IUGR and NR IUGR pregnancies as previously described [21]. A Gene Chip One-cycle Target Labeling Kit (Affymetrix, Santa Clara, CA, USA) was used to label total RNA, which was then hybridized to the Affymetrix GeneChip Bovine and Ovine Genome 1.0 ST Arrays. Hybridization quality was assessed using GCOS 1.4 (Affymetrix, Santa Clara, CA, USA). Hybridization probes for the Affymetrix GeneChip Bovine and Ovine Genome 1.0 ST Arrays (Affymetrix, Santa Clara, CA, USA) were prepared using 10 mg of total RNA and the One-Cycle Target Labeling and Control Reagent package (Affymetrix). The GeneChip Hybridization, Wash, and Stain Kit (Affymetrix, Santa Clara, CA, USA) and a Fluidic Station 450 (Affymetrix, Santa Clara, CA, USA) were used for the hybridization, wash, and staining process. All steps were carried out according to the manufacturer’s protocol. The processed arrays were scanned with a GeneChip Scanner 3000 (Affymetrix, Santa Clara, CA, USA). 

Array output was normalized via the robust multiarray method, and probe sets were filtered based on expression calls, as previously described [21,22]. Data analysis was conducted using the GeneSpring GX Software (Agilent Technologies) using ANOVA (*p* ≤ 0.05) with a Benjamini and Hochberg false discovery rate multiple test correction to determine differentially expressed genes in placentomes from NR non-IUGR and NR IUGR pregnancies. 

### 2.3. Database for Annotation, Visualization, and Integrated Discovery

DAVID version 6.7 (http://david.abcc.ncifcrf.gov/home.jsp) facilitates the use of microarray gene lists to generate specific functional annotations of biological processes affected by treatment in microarray experiments [23,24,25]. DAVID was utilized, as previously described, to annotate biological themes in response to dietary treatment [25]. All differentially expressed genes identified were both significantly (*p* ≤ 0.05) and numerically (1.5-fold change or greater) different and homologous to a known and annotated human gene for use in the DAVID analysis. The background list utilized in the program included all genes assigned a human accession number that were present on the bovine or ovine oligo array. With Gene Ontology (GO) terms identified as biological mechanism, cellular component, and molecular function, along with protein domain and biochemical pathway membership, DAVID generated biological themes by grouping similar terms, ultimately creating functional annotation clusters associated with effects of dietary treatment [25].

### 2.4. cDNA Synthesis and qPCR Analyses

Synthesis of cDNA from total cellular RNA (2 µg) using random primers (Invitrogen, Carlsbad, CA, USA), oligo-dT primers, and SuperScript II Reverse Transcriptase (Invitrogen) was achieved as described previously [26]. Newly synthesized cDNA was acid-ethanol precipitated, re-suspended in 20 μL water at a dilution of 100 ng, and stored at −20 °C for qPCR analysis. Primer information is found in Table S1. Quantitative PCR analysis of mRNAs was performed using an ABI PRISM 7700 (Applied Biosystems, Foster City, CA, USA) with Power SYBR Green PCR Master Mix (Applied Biosystems) as the detector, according to manufacturer’s recommendations and using methods described previously [27]. Cycle parameters for qPCR were 50 °C for 2 min, 95 °C for 10 min, and then 95 °C for 15 s and 60 °C for 1 min for 40 cycles. Selected genes analyzed for microarray validation included: anterior gradient protein 2 homolog (*AGR2*), UDP-Gal:betaGlcNAc beta 1,3-galactosyltransferase, polypeptide 2 (*B3GALT2*), cell adhesion molecule 1 (*CADM1*), leukocyte antigen CD37 (*CD37*), T-lymphocyte activation antigen CD86 (*CD86*), C-X-C motif chemokine 10 (*CXCL10*), chemokine (C-X-C motif) ligand 14 (*CXCL14*), cathepsin S (*CTSS*), dihydropyrimidine dehydrogenase (*DPYD*), glycine amidinotransferase (L-arginine:glycine amidinotransferase) (*GATM*), histone deacetylase 11 (*HDAC11*), interleukin 12 receptor, beta 2 (*IL12RB2*), lipase, endothelial (*LIPG*), nucleoporin 210kDa (*NUP210*), solute carrier family 44, member 4 (*SLC44A4*), solute carrier organic anion transporter family, member 1C1 (*SLCO1C1*), secreted phosphoprotein 1 (S*PP1*), stanniocalcin 1 (*STC1*), and sulfatase 2 (*SULF2*). Primers were designed using Genebank sequences and are found in Supplemental Table S1.

Template input was optimized from serial dilutions of pooled placentomal cDNA for each gene to ensure that the amplification reaction achieved 95–105% efficiency, and dissociation curves were analyzed with each run to verify amplification of a single product. Final reactions for *CADM1*, *GATM*, *HDAC11*, *LIPG*, *SLC44A4* used 2 ng, *B3GALT2*, *CD37*, *CD86*, *CTSS*, *CXCL10*, *CXCL14*, *DPYD*, *IL12RB2*, *NUP210*, *SLCO1C1*, and *STC1* used 2.5 ng, *SPP1* used 5 ng, *SULF2* used 10 ng, and *AGR2* used 12.5 ng of input. Data were analyzed using 7200HT SDS software (version 2.3, Applied Biosystems). The relative quantification of gene expression across treatments was evaluated using the comparative CT method as previously described with beta actin (*ACTB)* used for normalization [27]. Statistical analysis of each gene compared NR non-IUGR and NR IUGR placentomes to validate the Affymetrix GeneChip Bovine and Ovine Genome 1.0 ST Arrays. Mean gene expression values from placentomes from control fed ewes have been included for informative comparisons only, and were not included in the statistical analysis.

### 2.5. Cloning of Partial cDNAs

Partial cDNAs were amplified by RT-PCR using placentomal total RNA, isolated from Day 125 of pregnancy, and specific primers (Supplemental Table S1) using methods described previously [25,28]. PCR amplification was conducted as follows for *CTSS, IL12RB2,* and *STC*: (1) 95 °C for 5 min; (2) 95 °C for 30 s; 58 °C for 30 s (for *CTSS,* and *IL12RB2*), and 60 °C (for *STC1*); and 72 °C for 30 s for 35 cycles; and (3) 72 °C for 7 min. The partial cDNAs of the correct predicted size were cloned into pCRII using a T/A Cloning Kit (Invitrogen) and the sequence of each verified using an ABI PRISM Dye Terminator Cycle Sequencing Kit and ABI PRISM automated DNA sequencer (Perkin-Elmer Applied Biosystems).

### 2.6. In Situ Hybridization

Localization of mRNAs in the ovine placentome was determined by radioactive in situ hybridization analysis as described previously [25,28]. Exposure times were as follows: three weeks for *CTSS* and *STC1*, and six weeks for *IL12RB2*. Images of representative fields were recorded under bright-field or dark-field illumination using a Nikon Ni-E motorized research microscope with Apochromat Lamda 4X, 10X, 20X and 40X objectives. 

### 2.7. Statistical Analysis

Data were subjected to least-squares analysis of variance using the General Linear Models procedures of the Statistical Analysis System (SAS Institute, Cary, NC, USA) and are presented as least-squares means with overall standard error of the mean (SE). There was no effect of fetal sex in the statistical model; therefore, it was removed from the statistical model. Differences in means were considered to be statistically significant when *p* ≤ 0.05, while *p* ≤ 0.10 was considered a tendency toward significance. Data from qPCR analyses for placentomes from NR non-IUGR and NR IUGR pregnancies were subjected to least-squares analysis of variance using the General Linear Model procedures of the Statistical Analysis System (SAS Institute, Cary, NC, USA).

## 3. Results

### 3.1. Microarray Analysis

To capitalize on our observed natural population variance in fetal weight in response to maternal NR, we conducted a gene expression array to identify novel genes in the placentomal transcriptome that regulate placental growth and/or function. Maternal weights and placentome characteristics as well as maternal and fetal nutrient levels for this study have been published elsewhere [15]. A summary of this approach identified 103 differentially expressed genes in placentomes from ewes having NR non-IUGR versus NR IUGR fetuses (Table 1). Within this set of differentially expressed genes, 15 genes were upregulated, and 88 genes were downregulated in placentomes from ewes having NR non-IUGR fetuses compared to those having IUGR fetuses.

### 3.2. Validation of Selected Genes

**Figure 1:**
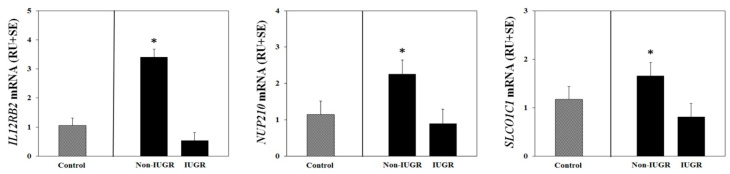
Select microarray-identified genes upregulated in nutrient restricted (NR) non-IUGR placetomes were validated using qPCR. Expression of *IL2RB2*, *NUP210*, and *SLCO1C1* mRNAs were greater (*p* < 0.05) in placentomes from NR non-IUGR fetuses compared to IUGR placentomes. Mean gene expression values from control ewes are included for informative comparisons only, and were not included in the statistical analysis. * Indicates *p* < 0.05 between NR Non-IUGR and NR IUGR placentomes.

**Figure 2:**
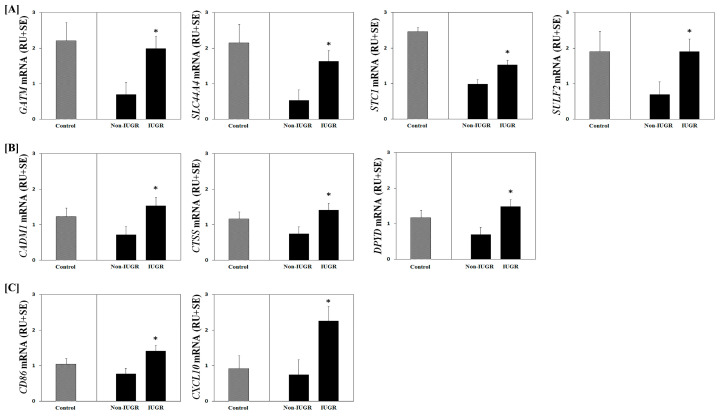
Select microarray-identified genes downregulated in NR non-IUGR placentomes were validated using qPCR. (**A**) *GATM, SLC44A4, STC1* and *SULF2* mRNA expression was greater (*p* < 0.05) in NR IUGR compared to NR non-IUGR placentomes. (**B**) *CADM1, CTSS,* and *DPYD* mRNA expression was greater (*p* < 0.05) in NR IUGR than NR non-IUGR placentomes. (**C**) *CD86* and *CXCL10* mRNA expression was greater (*p* < 0.05) in NR IUGR than NR non-IUGR placentomes. Mean gene expression values from control fed ewes have been included for informative comparisons only and were not included in the statistical analysis. Legend: RU, relative units. * Indicates *p* < 0.05 between NR Non-IUGR and NR IUGR placentomes.

A summary comparison of differentially expressed genes selected for validation of the microarray can be found in Table 2. Expression of *IL12RB2, NUP210,* and *SLCO1C1* mRNAs were higher (*p* < 0.05) in NR non-IUGR compared to NR IUGR placentomes (Figure 1). In contrast, *CADM1, CD86, CTSS, CXCL10, DPYD, GATM, SLC44A4, STC1*, and *SULF2* mRNA expression was increased (*p* < 0.05) in placentomes from ewes having NR IUGR fetuses compared to NR non-IUGR fetuses (Figure 2). These results validate gene expression based on transcriptional profiling analyses and indicate that genes are differentially expressed in NR non-IUGR compared with NR IUGR placentomes.

**Figure 3:**
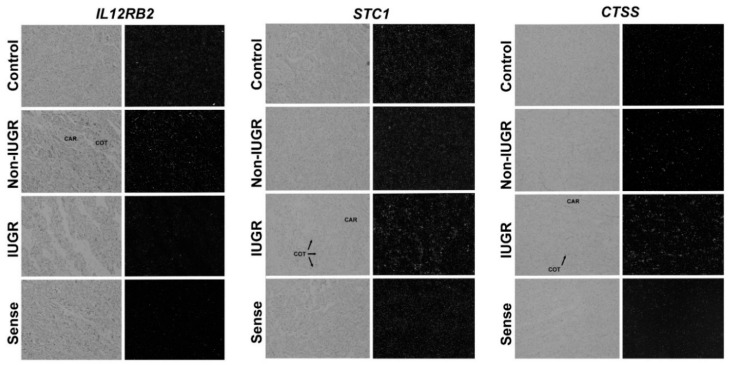
Localization of IL12RB2, STC1, and CTSS mRNA in placentomes of control-fed, NR non-IUGR, and NR IUGR pregnancies. Expression of IL12RB2 mRNA was weak in the placentomes of control or NR IUGR pregnancies. Expression of IL12RB2 mRNA was detected in scattered cells throughout the carunclular stroma of NR non-IUGR placentomes. The relative abundance of STC1 and CTSS mRNAs was greater in the cotyledonary tissue of NR IUGR placentomes as compared to NR non-IUGR or controls. Expression of STC1 mRNA was detected in a diffuse pattern throughout the cotyledonary tissue, while CTSS appeared to be more abundant at the fetal-maternal interface of the cotyledon and caruncle. CAR denotes caruncle and COT denotes cotyledon. All photomicrographs are shown at the same width of field (420 µm).

As determined by *in situ* hybridization, expression of *IL12RB2* mRNA was weak in the placentomes of control or NR IUGR pregnancies (Figure 3). However, the expression of *IL12RB2* mRNA was detected in scattered cells throughout the caruncular stroma of the placentomes of NR non-IUGR pregnancies. The relative abundance of *STC1* and *CTSS* mRNAs was greater in the cotyledonary tissue of NR IUGR placentomes as compared to that of NR non-IUGR or controls (Figure 3). Expression of *STC1* mRNA was detected in a diffuse pattern throughout the cotyledonary tissue, while *CTSS* appeared to be more abundant at the interface of the cotyledon and caruncle.

### 3.3. Bioinformatics

DAVID bioinformatic analyses were performed to identify biological processes potentially regulating the differential rates of placental growth and/or function between NR non-IUGR and NR IUGR ewes. DAVID analysis of the 15 genes upregulated in NR non-IUGR pregnancies identified three weakly enriched functional annotation clusters, which were associated with biological terms such as transmembrane region, integral to membrane, intrinsic to membrane, receptor, cell surface receptor linked signal transduction, signal peptide, alternative splicing, and splice variant (Table 3). 

Conversely, thirty-three enriched clusters were identified by DAVID analysis of the 88 downregulated genes from NR non-IUGR placentomes. The 10 most highly enriched clusters are presented in Table 4. Interestingly, of the ten most enriched clusters, two were associated with response to nutrients, while five were associated with immune response. Clusters associated with a response to nutrients featured biological terms, such as: response to nutrient, response to extracellular stimulus, amino acid transport, amine transport, amino acid transmembrane transporter activity, carboxylic acid transport, organic acid transport, and amine transmembrane transporter activity. Those clusters related to immune responses featured biological terms, such as: positive regulation of immune response, immune effector process, immunoglobulin-like, immunoglobulin domain, activation of immune response, complement activation, activation of plasma proteins involved in acute inflammatory response, humoral immune response, lymphocyte mediated immunity, adaptive immune response, and leukocyte mediated immunity.

## 4. Discussion

Microarray analysis of placentomes from NR ewes identified novel candidate genes that may regulate development and/or function of the placentome, giving rise to differing rates of fetal growth. Placentomal genes expressed later in gestation, in this case gestational Day 125, are likely indicative of either prior changes in placental development, which set a pathway(s) in motion, or factors regulating the substantial rate of fetal growth that occurs during the final trimester. Indeed, previous studies using models of nutrient restriction in pregnant ewes have shown that throughout gestation, genes such as nutrient transporters [29,30] and angiogenic factors [11,12,31] are essential in regulating proper fetal development. Likewise, data from various pregnancy models in livestock, humans, and mice illustrate the importance of placental development and gene function on fetal development [9,32,33].

Previous work from our laboratory using the same total caloric nutrient restriction model has shown that the expression of various nutrient transporters is upregulated in placentomes from non-IUGR fetuses compared to their IUGR counterparts [15]. This, along with the increased placental and fetal weights in non-IUGR pregnancies of NR ewes, led to the hypothesis that adaptive mechanisms exist in a subset of nutrient restricted ewes to maintain normal fetal growth despite limited maternal nutrient availability. However, results from the present study suggest that enhanced fetal growth in NR ewes is associated with an altered immune response, rather than solely a compensatory up-regulation of genes involved in placental development and function. Furthermore, the specific nutrient transporters that were upregulated in the non-IUGR compared to IUGR placentomes from NR ewes previously, were not detected in this microarray [15]. This is likely due to the selection criteria of a 1.5-fold change or greater, as many of the previously discussed genes exhibited smaller fold changes. While these select nutrient transporters significantly impact placental function and fetal development, we increased the stringency of our selection criteria of this microarray in order to elucidate novel genes influencing placental development and function.

DAVID bioinformatic analysis of the 15 genes up-regulated in non-IUGR pregnancies from NR ewes identified only three functional annotation clusters. Those clusters featured GO terms such as integral to membrane, intrinsic to membrane, and cell surface receptor linked signal transduction. Select genes found in those clusters included *IL12Rβ2*, *NUP210*, *SLCO1C1*, *β3GALT2*, and *LIPG*.

The interleukin-12 receptor is known to be expressed primarily on natural killer (NK) and activated T cells, with the β2 subunit being restricted to Th1 lymphocytes [34,35,36]. Thus, when acting with its ligand Il-12, IL12Rβ2 may mediate differentiation of Th1 lymphocytes [34,37]. During implantation and pregnancy, there appears to be a shift towards a greater population of Th2 lymphocytes at the maternal-fetal interface [38]. Th1 lymphocytes produce cytokines that can compromise pregnancy, while cytokines produced by Th2 lymphocytes inhibit inflammatory Th1 responses at the maternal-fetal interface to allow implantation and pregnancy to occur [38]. While there is an up-regulation of *IL12Rβ2* mRNA in the placentomes of NR non-IUGR pregnancies, the present study did not investigate the amount of IL-12 in placentomes, or presence of other IL-12 receptors, and further work is needed to fully elucidate the implications of the increased *IL12Rβ2* mRNA in the placentomes of NR non-IUGR pregnancies.

Approximately 30 proteins known as nucleoporins serve as building blocks for nuclear pore complexes (NPCs) at fusion sites between the inner and outer nuclear membranes. NUP210 is one of only three integral membrane proteins in the various components of the NPC [39]. The complete function of NUP210 is not clear. However, in mice it has been shown to be involved in epithelial cell development in various organs, and is required for myogenic and neuronal differentiation, serving a role in cell fate determination and regulation of gene expression [39,40]. Therefore, it is possible that NUP210 in the placentomes during late gestation regulate cell fate determination and expression of genes that allow this subpopulation of ewes to produce NR non-IUGR fetuses. To our knowledge, this is the first study showing *NUP210* mRNA expression in the placenta and further work to assess localization is still needed.

The thyroid hormones (TH), triiodothyronine (T_3_) and thyroxine (T_4_), are imperative to normal in utero growth and development as they promote growing fetal mass and terminal tissue differentiation [41]. The organic anion transporter SLCO1C1 (also known as OATP1C1 and OATP14) is primarily expressed at the blood-brain barrier for transport of T_4_ to the developing brain, although it is also expressed in human Leydig cells [42,43]. Interestingly, SLCO1C1 was recently found to be strongly expressed in the villous stroma of the rat placenta [42]. Permeability of the placenta to TH is partly dependent on species and placental type. Humans and rodents, having a hemochorial placenta, are relatively permeable to T_3_ and T_4_, while livestock, possessing epitheliochorial and synepitheliochorial placentas, are thought to be seemingly impermeable to maternal THs [41]. Moreover, concentrations of TH are low in human IUGR infants and in IUGR offspring from placental insufficiency and NR animal models [41,44,45].

In the present study, 88 genes were downregulated in placentomes from NR non-IUGR versus NR IUGR conceptuses. Interestingly, a number of these genes appeared to display similar patterns of expression between the NR IUGR and control placentomes, with these genes being downregulated in the NR non-IUGR pregnancies. DAVID analysis of these genes revealed 33 functional annotation clusters. Not surprisingly, the most significant cluster featured the GO terms: response to nutrient levels, response to nutrient, and response to extracellular stimulus. However, of the 10 most significant annotation clusters identified, half were related to immune response, with GO terms such as positive regulation of immune response, immune effecter process, activation of immune response, humoral immune response, lymphocyte mediated immunity, leukocyte mediated immunity, and adaptive immune response. 

Establishment and maintenance of pregnancy in all mammalian species involves an intricate balance of immune cells, particularly a balance of pro- and anti-inflammatory cytokines regulated by the maternal immune system, at the maternal-placental interface [46]. This balance is largely regulated by the presence of progesterone, which allows for local inhibition of immune responses at the maternal-placental interface without resulting in systemic immunosuppression [47]. In a clinical setting, increases in inflammatory cytokines, such as TNF-α, and the chemokine IL-8, are seen in placentas from IUGR pregnancies [48,49]. Umbilical artery ligation in sheep induces a fetoplacental inflammatory response, characterized by increased pro-inflammatory cytokines, and ultimately results in IUGR at Day 116 of gestation [50]. Overall, work illustrating immune responses in the placentas of IUGR pregnancies are limited. Furthermore, to our knowledge, data on the immunological profile of the placenta in response to maternal nutrient restriction is lacking and the present study presents novel genes regulating immune responses within the placenta of nutrient restricted pregnancies, which result in IUGR.

The rate-limiting enzyme for creatine synthesis, glycine amidinotransferase (GATM), decreases production of proinflammatory nitric oxide by competing with the inducible form of nitric oxide synthase for the amino acid arginine [51]. Additionally, *GATM* is an imprinted gene in human and mouse placentas [52,53], with expression being exclusively from the maternal allele in extraembryonic tissues of mice [53]. Importantly, a genome-wide survey discovered increased expression of *GATM* of placentas from women that gave birth to an IUGR fetus [54]. Expression of *GATM* is also seen in bovine endometrial CD14^+^ cells, potentially serving roles characteristic of M2 activated macrophages, such as tissue remodeling and immune regulation for promoting pregnancy [55]. It is hypothesized that expression of *GATM* in the placenta for production of phosphocreatine might reduce the impact of sudden high-energy demands from the fetus on the gestating dam [53]. 

Stanniocalcin 1 (STC1) is a glycoprotein that regulates calcium and phosphate homeostasis in a paracrine manner in the kidney and intestine [56,57]. During pregnancy in sheep, *STC1* is involved in regulation of placental and fetal growth and differentiation with expression appearing in the endometrial glands on Day 18 of gestation and increasing until Day 80 [58]. Levels of *STC1* mRNA remain elevated in the uterine glands through gestational Day 120 and are associated with the secretion of STC1 protein into the glands and transport via the placental areolae into fetal circulation and allantoic fluid [58]. In a study by Song et al. [58], the expression of *STC1* mRNA was not detected in the placentomes of ewes from Days 30, 40, 60, 80, 100, 120, or 140 of gestation. However, in the present study we detected low levels of *STC1* mRNA in the placentome at Day 125 of gestation. It is probable that the stress of undernutrition stimulates up-regulation of *STC1* mRNA in the NR IUGR compared the NR non-IUGR pregnancies, but its overall function in the placentome is still unclear.

Uterine remodeling is initiated during the early stages of gestation and continues until parturition to ensure proper implantation and placentation for normal fetal development. This tissue remodeling is partially supported by the degradation of the extracellular matrix and catabolism of intracellular hormones stimulated by a group of peptidases known as cathepsins [59,60]. Expression of various cathepsins has been detected in ovine uteroplacental tissues throughout gestation. Cathepsin S, in particular, was found in both the intercaruncular endometrium, as well as the placentome through Day 120 of gestation [59]. More specifically, the expression of *CTSS* mRNA increased in the stratum compactum stroma, but declined in the caruncular stroma during gestation [59]. Our data indicate that mRNA levels of *CTSS* are increased in NR IUGR compared to NR non-IUGR placentomes, with *CTSS* being localized in the cotyledonary villi and being more abundant at the fetal-maternal interface of the placentome at gestational Day 125. An increase in *CTSS* mRNA in the placentomes of NR IUGR pregnancies during late gestation may represent a failed attempt to enhance vascular or tissue remodeling to improve placental function. Alternatively, a decrease in *CTSS* mRNA in the placentomes of NR non-IUGR pregnancies may indicate increased remodeling at the fetal-maternal interface of NR IUGR placentomes to enhance nutrient transfer to the rapidly growing fetus at this point in gestation.

In addition to its role as a cysteine protease, CTSS is also essential to major histocompatibility complex (MHC) class II antigen presentation and proteolysis [61,62]. CTSS-deficient mice (*CTSS*^-/-^) have normal populations of B and T cells, but have an impaired ability to degrade the invariant chain (Ii), which is necessary for MHC class II molecules to acquire antigenic peptides and undergo peptide binding [63,64]. While the expression of MHC class II molecules in the placentome is not fully understood, there is expression of MHC class I during late gestation, around the time of parturition [65,66]. Additionally, parturition in cattle is associated with increased apoptosis, degradation of the extracellular matrix, and an innate immune response [67]. These physiological processes and complexes at late gestation align with the genes and functional annotation clusters discovered in the present study, but the role of CTSS in the placentomes of NR ewes with IUGR fetuses is not clear and warrants further investigation.

Epigenetic alterations, such as DNA methylation, during fetal development can profoundly influence the susceptibility of offspring to postnatal diseases through fetal programming. Therefore, it is a common clinical practice for gestating mothers to be supplemented with methyl donors such as folate and choline [7]. Previous work in our laboratory with ewes revealed that the sodium-dependent choline transporter, SLC44A4, is up-regulated in the endometrium during early pregnancy, and in response to exogenous progesterone [25]. Studies in rodents and humans have shown that a deficiency in choline during early pregnancy can lead to neural tube defects and other brain defects during postnatal life [68]. Thus, an increase in the expression of *SLC44A4* mRNA in IUGR compared to non-IUGR placentomes could be an attempt to prevent choline deficiency in IUGR lambs in response to NR. However, levels of choline were not measured in these studies.

## 5. Conclusions

Results of the present study illustrate that placentomal gene expression in late gestation is indicative of an altered immune response, which is associated with enhanced fetal growth, in a subpopulation of NR ewes. This altered immune response may work in conjunction with increased expression of certain nutrient transporters to promote fetal nutrient availability to enhance fetal growth in non-IUGR pregnancies from NR ewes. Future studies are necessary to investigate the immune cell profile and immunological forces at play within the placentae of compromised and adaptive pregnancies. 

## Figures and Tables

**Table 1:** Placentomal mRNA expression for selected genes identified using the microarray analysis in nutrient restricted (NR) non-intrauterine growth restriction (non-IUGR) compared to NR IUGR pregnancies (*p* < 0.05).

Gene Symbol	Gene Name	Fold Change	Gene Symbol	Gene Name	Fold Change
*A4IFS4*	Pregnancy-associated glycoprotein 16	2.11	*LBP*	Lipopolysaccharide binding protein	−1.68
*ADH6*	Alcohol dehydrogenase 6 (class V)	−1.99	*LIPG*	Endothelial lipase	2.00
*AGR2*	Anterior gradient protein 2 homolog	−2.32	*Mamu-DRA*	Mamu class II histocompatibility antigen, DR alpha chain	−1.78
*AGTR2*	Angiotensin II receptor, type 2	−1.89	*MCEMP1*	Mast cell-expressed membrane protein 1	1.70
*AKR1C1*	Aldo-keto reductase family 1 member C1	−2.13	*MICB*	MHC class I polypeptide-related sequence B	−1.75
*ALDH1A1*	Aldehyde dehydrogenase 1 family, member A1	−1.54	*MILR1*	Allergin-1	−1.57
*B3GALT2*	Beta-1,3-galactosyltransferase 2	1.57	*MIR186*	MicroRNA mir-186	−2.16
*B3GNT3*	Beta-1,3-N-acetylglucosaminyltransferase BGnT-3	1.74	*MIR29A*	MicroRNA mir-29a	1.53
*BCL2L15*	Bcl-2-like protein 15	−2.17	*MIR329B*	MicroRNA mir-329b	−1.55
*BOLA-DRA*	Major histocompatibility complex, class II, DR alpha	−1.69	*MS4A8A*	Membrane-spanning 4-domains subfamily A member 8A	−1.72
*BOLA-DRB3*	Major histocompatibility complex, class II, DRB3	−1.65	*MSLN*	Mesothelin	−1.56
*C1QB*	Complement C1q subcomponent subunit B	−1.67	*MSR1*	Macrophage scavenger receptor types I and II	−1.65
*C4ORF19*	Uncharacterized protein C4orf19	−1.60	*MUC16*	Mucin-16	−1.64
*CADM1*	Cell adhesion molecule 1	−1.58	*NUP210*	Nuclear pore membrane glycoprotein 210	1.77
*CCKN*	Cholecystokinin Precursor	1.77	*OCIAD2*	OCIA domain-containing protein2	−1.55
*CD200R1*	Cell surface glycoprotein CD200 receptor 1	−1.51	*OGN*	Mimecan	−1.88
*CD37*	Leukocyte antigen CD37	−1.59	*OR52E1*	Olfactory receptor 52E1	1.53
*CD86*	T-lymphocyte activation antigen CD86	−1.51	*OSTP*	Osteopontin Precursor	−1.52
*CFD*	Complement factor D	−1.55	*P2RY12*	P2Y purinoceptor 12	−1.61
*CH3L1*	Chitinase 3-like protein 1 Precursor	−2.13	*PAM*	Peptidyl-glycine alpha-amidating monooxygenase	−1.58
*CHRM2*	Muscarinic acetylcholine receptor M2	−1.53	*PDCD2L*	Programmed cell death protein 2-like	1.83
*CP*	Ceruloplasmin (ferroxidase)	−2.25	*PDZK1IP1*	PDZK1-interacting protein 1	−1.61
*CPE*	Carboxypeptidase E	−1.60	*PEBP4*	Phosphatidylethanolamine-binding protein 4	−2.46
*CR2*	Complement receptor type 2	−2.93	*PRR15*	Proline-rich protein 15	−1.70
*CST6*	Cystatin-M	−1.59	*QSOX1*	Quiescin Q6 sulfhydryl oxidase 1	−1.63
*CTSS*	Cathepsin S	−1.54	*RBP4*	Retinol-binding protein 4	−1.59
*CXCL10*	C-X-C motif chemokine 10	−1.85	*RNASE6*	Ribonuclease K6	−1.67
*CXCL14*	C-X-C motif chemokine 14	−2.17	*S100A7*	S100 calcium binding protein A7	−1.51
*CYP26A1*	Cytochrome P450, family 26, subfamily A, polypeptide 1	−1.57	*SAA3*	Serum amyloid A 3	−2.08
*CYP4F22*	Cytochrome P450 4F22	1.51	*SDS*	L-serine dehydratase/L-threonine deaminase	−1.66
*DLK1*	Protein delta homolog 1	−1.62	*SERPINE2*	Glia-derived nexin	−1.62
*DPYD*	Dihydropyrimidine dehydrogenase	−1.51	*SESN3*	Sestrin-3	−1.61
*EHF*	ETS homologous factor	−1.60	*SIGLEC1*	Sialic acid binding Ig-like lectin 1, sialoadhesin	−1.54
*EMP1*	Epithelial membrane protein 1	−1.53	*SLC1A1*	Excitatory amino acid transporter3	−1.72
*EVI2B*	Ecotropic Viral Integration Site 2B	−1.59	*SLC26A3*	Chloride anion exchanger	−1.82
*FAM134B*	Reticulophagy Regulator 1	−1.71	*SLC37A2*	Sugar phosphate exchanger 2	−1.68
*FCGR3*	Low affinity immunoglobulin gamma Fc region receptor III	−1.61	*SLC44A4*	Choline transporter-like protein 4	−2.19
*FGFR1*	Fibroblast growth factor receptor 1	−1.60	*SLC7A2*	Low affinity cationic amino acid transporter 2	−2.22
*FOLR2*	Folate receptor beta	−1.54	*SLC7A9*	B(0,+)-type amino acid transporter1	−1.82
*GATA6*	Transcription factor GATA-6	−1.66	*SLCO1C1*	Solute carrier organic anion transporter family member 1C1	1.78
*GATM*	Glycine amidinotransferase, mitochondrial	−2.01	*SPP1*	Secreted phosphoprotein 1	−1.56
*GHR*	Growth hormone receptor	−1.55	*STC1*	Stanniocalcin-1	−1.91
*GPR115*	Probable G-protein coupled receptor 115	−1.66	*SULF2*	Extracellular sulfatase Sulf-2	−1.64
*GPR151*	Probable G-protein coupled receptor 151	2.20	*TC2N*	Tandem C2 domains nuclear protein	−2.26
*GRM7*	Metabotropic glutamate receptor 7	−2.66	*TFEC*	Transcription factor EC	−1.51
*HDAC11*	Histone deacetylase 11	1.58	*TFPI2*	Tissue factor pathway inhibitor 2	−1.64
*HTR4*	Serotonin 5-HTA receptor	−2.78	*THBS4*	Thrombospondin-4	−1.62
*IL12RB2*	Interleukin-12 receptor subunit beta-2	1.88	*TIMD4*	T-cell immunoglobulin and mucin domain containing 4	−1.59
*INHBA*	Inhibin, beta A	−1.85	*VNN1*	Pantetheinase	−1.53
*KLF5*	Krueppel-like factor 5	−1.54	*WNT11*	Protein Wnt-11	−1.81
*KNG2*	Kininogen-2	−1.68			

**Table 2:** Comparison of mRNA expression for selected genes identified using microarray or qPCR analyses in NR non-IUGR versus NR IUGR placentomes.

Gene Symbol	Microarray Fold Change ^a^	qPCR Fold Change	qPCR *p*-Value
*AGR2*	−2.32	−2.69	0.05
*B3GALT2*	1.57	1.67	0.09
*CADM1*	−1.58	−2.14	0.03
*CD37*	−1.59	−1.73	0.07
*CD86*	−1.51	−1.84	0.01
*CTSS*	−1.54	−1.88	0.03
*CXCL10*	−1.85	−3.01	0.03
*CXCL14*	−2.17	−2.79	0.12
*DPYD*	−1.51	−2.12	0.03
*GATM*	−2.01	−2.84	0.02
*HDAC11*	1.58	1.85	0.17
*IL12RB2*	1.88	6.40	0.00
*LIPG*	2.00	2.02	0.28
*NUP210*	1.77	2.50	0.03
*SLC44A4*	−2.19	−3.07	0.03
*SLCO1C1*	1.78	2.04	0.05
*SPP1*	−1.56	−3.41	0.09
*STC1*	−1.91	−1.54	0.03
*SULF2*	−1.64	−2.74	0.03

^a^ Microarray fold-changes are significant (*p* < 0.05).

**Table 3:** Functional annotation clusters of biological terms representing upregulated genes in NR non-IUGR compared to NR IUGR placentomes.

Annotation Cluster ^a^	Enrichment Score ^b^	Biological Terms ^c^
1	1.27	Transmembrane region (7); Transmembrane (7); GO:0016021 ~ integral to membrane (7); GO:0031224 ~ intrinsic to membrane (7)
2	0.51	Receptor (3); GO:0007166 ~ cell surface receptor linked signal transduction (3); Topological domain: Extracellular (3)
3	0.15	Signal (3); Signal peptide (3); Alternative splicing (3); Splice variant (3)

^a^ The three most significant annotation clusters identified from the gene list submitted for analysis through DAVID. ^b^ The enrichment score ranks the significance of each annotation cluster based on the relatedness of the terms and the genes associated with them. ^c^ This column summarizes the biological terms in the annotation clusters. The gene ontology (GO) terms were gathered based on the known annotation of the submitted genes with respect to biological process, cellular component, and molecular function as well as biological pathway membership and protein domains. The number in parentheses indicates the number of differentially expressed genes contributing to the clustered term.

**Table 4:** Functional annotation clusters of biological terms representing downregulated genes in NR non-IUGR compared to NR IUGR placentomes.

Annotation Cluster ^a^	Enrichment Score ^b^	Biological Terms ^c^
1	3.40	GO:0031667 ~ response to nutrient levels (7); GO:0007584 ~ response to nutrient (6); GO:0009991 ~ response to extracellular stimulus (7);
2	2.01	GO:0050778 ~ positive regulation of immune response (6); GO:0002252 ~ immune effector process (5); GO:0048584 ~ positive regulation of response to stimulus (6); Immune response (5)
3	2.01	Transmembrane region (32); Transmembrane (32); Membrane (37); GO:0031224 ~ intrinsic to membrane (36); GO:0016021 ~ integral to membrane (33)
4	1.91	GO:0005624 ~ membrane fraction (11); GO:0005626 ~ insoluble fraction (11); GO:0000267 ~ cell fraction (12)
5	1.67	Ig-like V-type (5); CD80-like, immunoglobulin C2-set (3); IG (4); Immunoglobulin subtype (4)
6	1.66	GO:0006865 ~ amino acid transport (4); GO:0015837 ~ amine transport (4); GO:0015171 ~ amino acid transmembrane transporter activity (3); GO:0046942 ~ carboxylic acid transport (4); GO:0015849 ~ organic acid transport (4); GO:0005275 ~amine transmembrane transporter activity (3)
7	1.62	Immunoglobulin-like (7); Immunoglobulin-like fold (7); Immunoglobulin domain (6)
8	1.61	GO:0042803 ~ protein homodimerization activity (6); GO:0046983 ~ protein dimerization (7); GO:0042802~identical protein binding (7)
9	1.54	GO:0051605 ~ protein maturation by peptide bond cleavage (4); GO:0002253 ~ activation of immune response (4); GO:0016485 ~ protein processing (4); GO:0006956 ~ complement activation (3); GO:0002541 ~ activation of plasma proteins involved in acute inflammatory response (3); GO:0051604 ~ protein maturation (4); Innate immunity (3); GO:0006959 ~ humoral immune response (3); Complement and coagulation cascades (3); GO:0006508 ~ proteolysis (5)
10	1.31	GO:0002449 ~ lymphocyte mediated immunity (3); GO:0002250 ~ adaptive immune response (3); GO:0002460 ~ adaptive immune response based on somatic recombination of immune receptors built from immunoglobulin superfamily domains (3); GO:0002443 ~ leukocyte mediated immunity (3)

^a^ The 10 most significant annotation clusters identified from the gene list submitted for analysis through DAVID. ^b^ The enrichment score ranks the significance of each annotation cluster based on the relatedness of the terms and the genes associated with them. ^c^ This column summarizes the biological terms in the annotation clusters. The gene ontology (GO) terms were gathered based on the known annotation of the submitted genes with respect to biological process, cellular component, and molecular function; as well as biological pathway membership and protein domains. The number in parentheses indicates the number of differentially expressed genes contributing to the clustered term.

## Supplementary Materials

The following are available online at https://www.mdpi.com/2073-4425/11/9/1031/s1, Table S1: Primer sequences for qPCR and ISH analyses.

## References

[1] Wallace J.M., Regnault T.R., Limesand S.W., Hay W.W., Anthony R.V. (2005). Investigating the causes of low birth weight in contrasting ovine paradigms. J. Physiol..

[2] Wu G., Bazer F.W., Wallace J.M., Spencer T.E. (2006). Board-invited review: Intrauterine growth retardation: Implications for the animal sciences. J. Anim. Sci..

[3] Bell A.W., Ehrhardt R.A. (2002). Regulation of placental nutrient transport and implications for fetal growth. Nutr. Res. Rev..

[4] NRC, N.R.C (1985). Nutrient Requirements for Sheep.

[5] Marsal K. (2002). Intrauterine growth restriction. Curr. Opin. Obstet. Gynecol..

[6] Barker D.J.P. (2007). The origins of the developmental origins theory. J. Intern. Med..

[7] Wu G., Bazer F.W., Cudd T.A., Meininger C.J., Spencer T.E. (2004). Maternal nutrition and fetal development. J. Nutr..

[8] Ford S.P., Long N.M. (2011). Evidence for similar changes in offspring phenotype following either maternal undernutrition or overnutrition: Potential impact on fetal epigenetic mechanisms. Reprod. Fertil. Dev..

[9] Bell A.W., Ehrhardt R.A. (1998). Placental Regulation of Nutrient Partitioning During Pregnancy. Nutr. Reprod..

[10] Heasman L., Clarke L., Stephenson T.J., Symonds M.E. (1999). The influence of maternal nutrient restriction in early to mid-pregnancy on placental and fetal development in sheep. Proc. Nutr. Soc. India.

[11] Reynolds L.P., Borowicz P.P., Vonnahme K.A., Johnson M.L., Grazul-Bilska A.T., Wallace J.M., Caton J.S., Redmer D.A. (2005). Animal models of placental angiogenesis. Placenta.

[12] Reynolds L.P., Redmer D.A. (2001). Angiogenesis in the placenta. Biol. Reprod..

[13] Owens J.A., Falconer J., Robinson J.S. (1986). Effect of restriction of placental growth on umbilical and uterine blood flows. Am. J. Physiol..

[14] Long N.M., Vonnahme K.A., Hess B.W., Nathanielsz P.W., Ford S.P. (2009). Effects of early gestational undernutrition on fetal growth, organ development, and placentomal composition in the bovine. J. Anim. Sci..

[15] Edwards A.K., McKnight S.M., Askelson K., McKnight J.R., Dunlap K.A., Satterfield M.C. (2020). Adaptive responses to maternal nutrient restriction alter placental transport in ewes. Placenta.

[16] Cross J.C., Mickelson L. (2006). Nutritional influences on implantation and placental development. Nutr. Rev..

[17] Mellor D.J. (1983). Nutritional and placental determinants of foetal growth rate in sheep and consequences for the newborn lamb. Br. Vet. J..

[18] Lassala A., Bazer F.W., Cudd T.A., Datta S., Keisler D.H., Satterfield M.C., Spencer T.E., Wu G. (2010). Parenteral administration of L-arginine prevents fetal growth restriction in undernourished ewes. J. Nutr..

[19] Borowicz P.P., Arnold D.R., Johnson M.L., Grazul-Bilska A.T., Redmer D.A., Reynolds L.P. (2007). Placental growth throughout the last two thirds of pregnancy in sheep: Vascular development and angiogenic factor expression. Biol. Reprod..

[20] Sandoval C., Lambo C.A., Beason K., Dunlap K.A., Satterfield M.C. (2020). Effect of maternal nutrient restriction on skeletal muscle mass and associated molecular pathways in SGA and Non-SGA sheep fetuses. Domest. Anim. Endocrinol..

[21] Spencer T.E., Forde N., Dorniak P., Hansen T.R., Romero J.J., Lonergan P. (2013). Conceptus-derived prostaglandins regulate gene expression in the endometrium prior to pregnancy recognition in ruminants. Reproduction.

[22] Irizarry R.A., Hobbs B., Collin F., Beazer-Barclay Y.D., Antonellis K.J., Scherf U., Speed T.P. (2003). Exploration, normalization, and summaries of high density oligonucleotide array probe level data. Biostatistics.

[23] Dennis G., Sherman B.T., Hosack D.A., Yang J., Gao W., Lane H.C., Lempicki R.A. (2003). DAVID: Database for Annotation, Visualization, and Integrated Discovery. Genome Biol..

[24] Huang D.W., Sherman B.T., Lempicki R.A. (2009). Systematic and integrative analysis of large gene lists using DAVID bioinformatics resources. Nat. Protoc..

[25] Satterfield M.C., Song G., Kochan K.J., Riggs P.K., Simmons R.M., Elsik C.G., Adelson D.L., Bazer F.W., Zhou H., Spencer T.E. (2009). Discovery of candidate genes and pathways in the endometrium regulating ovine blastocyst growth and conceptus elongation. Physiol. Genom..

[26] Stewart M.D., Johnson G.A., Gray C.A., Burghardt R.C., Schuler L.A., Joyce M.M., Bazer F.W., Spencer T.E. (2000). Prolactin receptor and uterine milk protein expression in the ovine endometrium during the estrous cycle and pregnancy. Biol. Reprod..

[27] Satterfield M.C., Hayashi K., Song G., Black S.G., Bazer F.W., Spencer T.E. (2008). Progesterone regulates FGF10, MET, IGFBP1, and IGFBP3 in the endometrium of the ovine uterus. Biol. Reprod..

[28] Spencer T.E., Stagg A.G., Joyce M.M., Jenster G., Wood C.G., Bazer F.W., Wiley A.A., Bartol F.F. (1999). Discovery and characterization of endometrial epithelial messenger ribonucleic acids using the ovine uterine gland knockout model. Endocrinology.

[29] Dunlap K., Brown J., Keith A., Satterfield M. (2015). Factors controlling nutrient availability to the developing fetus in ruminants. J. Animal. Sci. Biotechnol..

[30] Ma Y., Zhu M.J., Uthlaut A.B., Nijland M.J., Nathanielsz P.W., Hess B.W., Ford S.P. (2011). Upregulation of growth signaling and nutrient transporters in cotyledons of early to mid-gestational nutrient restricted ewes. Placenta.

[31] Redmer D.A., Aitken R.P., Milne J.S., Reynolds L.P., Wallace J.M. (2005). Influence of maternal nutrition on messenger RNA expression of placental angiogenic factors and their receptors at midgestation in adolescent sheep. Biol. Reprod..

[32] Reynolds L.P., Caton J.S., Redmer D.A., Grazul-Bilska A.T., Vonnahme K.A., Borowicz P.P., Luther J.S., Wallace J.M., Wu G., Spencer T.E. (2006). Evidence for altered placental blood flow and vascularity in compromised pregnancies. J. Physiol..

[33] Lang U., Baker R.S., Braems G., Zygmunt M., Kunzel W., Clark K.E. (2003). Uterine blood flow--a determinant of fetal growth. Eur. J. Obstet. Gynecol. Reprod. Biol..

[34] Meng X., Guo A., Gong W., Jia W., Luo X., Zhai J., Dou Y., Cai X. (2012). Molecular characterization, tissue distribution and expression analysis of interleukin-12 receptor beta2 chain in sheep. Gene.

[35] Rogge L., Barberis-Maino L., Biffi M., Passini N., Presky D.H., Gubler U., Sinigaglia F. (1997). Selective expression of an interleukin-12 receptor component by human T helper 1 cells. J. Exp. Med..

[36] Rogge L., Papi A., Presky D.H., Biffi M., Minetti L.J., Miotto D., Agostini C., Semenzato G., Fabbri L.M., Sinigaglia F. (1999). Antibodies to the IL-12 receptor beta 2 chain mark human Th1 but not Th2 cells in vitro and in vivo. J. Immunol..

[37] Barbulescu K., Becker C., Schlaak J.F., Schmitt E., Meyer zum Buschenfelde K.H., Neurath M.F. (1998). IL-12 and IL-18 differentially regulate the transcriptional activity of the human IFN-gamma promoter in primary CD4+ T lymphocytes. J. Immunol..

[38] Wegmann T.G., Lin H., Guilbert L., Mosmann T.R. (1993). Bidirectional cytokine interactions in the maternal-fetal relationship: Is successful pregnancy a TH2 phenomenon?. Immunol. Today.

[39] D’Angelo M.A., Gomez-Cavazos J.S., Mei A., Lackner D.H., Hetzer M.W. (2012). A change in nuclear pore complex composition regulates cell differentiation. Dev. Cell.

[40] Olsson M., Ekblom M., Fecker L., Kurkinen M., Ekblom P. (1999). cDNA cloning and embryonic expression of mouse nuclear pore membrane glycoprotein 210 mRNA. Kidney Int..

[41] Forhead A.J., Fowden A.L. (2014). Thyroid hormones in fetal growth and prepartum maturation. J. Endocrinol..

[42] Sun Y.N., Liu Y.J., Zhang L., Ye Y., Lin L.X., Li Y.M., Yan Y.Q., Chen Z.P. (2014). Expression of organic anion transporting polypeptide 1c1 and monocarboxylate transporter 8 in the rat placental barrier and the compensatory response to thyroid dysfunction. PLoS ONE.

[43] Hagenbuch B. (2007). Cellular entry of thyroid hormones by organic anion transporting polypeptides. Best Pract. Res. Clin. Endocrinol. Metab..

[44] Kilby M.D., Verhaeg J., Gittoes N., Somerset D.A., Clark P.M., Franklyn J.A. (1998). Circulating thyroid hormone concentrations and placental thyroid hormone receptor expression in normal human pregnancy and pregnancy complicated by intrauterine growth restriction (IUGR). J. Clin. Endocrinol. Metab..

[45] Rae M.T., Rhind S.M., Kyle C.E., Miller D.W., Brooks A.N. (2002). Maternal undernutrition alters triiodothyronine concentrations and pituitary response to GnRH in fetal sheep. J. Endocrinol..

[46] Clark D.A., Arck P.C., Chaouat G. (1999). Why did your mother reject you? Immunogenetic determinants of the response to environmental selective pressure expressed at the uterine level. Am. J. Reprod. Immunol..

[47] Hansen P.J. (1998). Regulation of uterine immune function by progesterone--lessons from the sheep. J. Reprod. Immunol..

[48] Hahn-Zoric M., Hagberg H., Kjellmer I., Ellis J., Wennergren M., Hanson L.A. (2002). Aberrations in placental cytokine mRNA related to intrauterine growth retardation. Pediatr. Res..

[49] Bartha J.L., Romero-Carmona R., Comino-Delgado R. (2003). Inflammatory cytokines in intrauterine growth retardation. Acta Obstet. Gynecol. Scand. Suppl..

[50] Bertucci M.C., Loose J.M., Wallace E.M., Jenkin G., Miller S.L. (2011). Anti-inflammatory therapy in an ovine model of fetal hypoxia induced by single umbilical artery ligation. Reprod. Fertil. Dev..

[51] Bronte V., Zanovello P. (2005). Regulation of immune responses by L-arginine metabolism. Nat. Rev. Immunol..

[52] Yu Y., Singh U., Shi W., Konno T., Soares M.J., Geyer R., Fundele R. (2008). Influence of murine maternal diabetes on placental morphology, gene expression, and function. Arch. Physiol. Biochem..

[53] Sandell L.L., Guan X.J., Ingram R., Tilghman S.M. (2003). Gatm, a creatine synthesis enzyme, is imprinted in mouse placenta. Proc. Natl. Acad. Sci. USA.

[54] McMinn J., Wei M., Schupf N., Cusmai J., Johnson E.B., Smith A.C., Weksberg R., Thaker H.M., Tycko B. (2006). Unbalanced placental expression of imprinted genes in human intrauterine growth restriction. Placenta.

[55] Oliveira L.J., McClellan S., Hansen P.J. (2010). Differentiation of the endometrial macrophage during pregnancy in the cow. PLoS ONE.

[56] Yeung B.H., Law A.Y., Wong C.K. (2012). Evolution and roles of stanniocalcin. Mol. Cell. Endocrinol..

[57] Uuskula L., Mannik J., Rull K., Minajeva A., Koks S., Vaas P., Teesalu P., Reimand J., Laan M. (2012). Mid-gestational gene expression profile in placenta and link to pregnancy complications. PLoS ONE.

[58] Song G., Bazer F.W., Wagner G.F., Spencer T.E. (2006). Stanniocalcin (STC) in the endometrial glands of the ovine uterus: Regulation by progesterone and placental hormones. Biol. Reprod..

[59] Song G., Bazer F.W., Spencer T.E. (2007). Differential expression of cathepsins and cystatin C in ovine uteroplacental tissues. Placenta.

[60] Song G., Spencer T.E., Bazer F.W. (2005). Cathepsins in the ovine uterus: Regulation by pregnancy, progesterone, and interferon tau. Endocrinology.

[61] Riese R.J., Wolf P.R., Bromme D., Natkin L.R., Villadangos J.A., Ploegh H.L., Chapman H.A. (1996). Essential role for cathepsin S in MHC class II-associated invariant chain processing and peptide loading. Immunity.

[62] Riese R.J., Mitchell R.N., Villadangos J.A., Shi G.P., Palmer J.T., Karp E.R., De Sanctis G.T., Ploegh H.L., Chapman H.A. (1998). Cathepsin S activity regulates antigen presentation and immunity. J. Clin. Investig..

[63] Shi G.P., Villadangos J.A., Dranoff G., Small C., Gu L., Haley K.J., Riese R., Ploegh H.L., Chapman H.A. (1999). Cathepsin S required for normal MHC class II peptide loading and germinal center development. Immunity.

[64] Hsieh C.S., deRoos P., Honey K., Beers C., Rudensky A.Y. (2002). A role for cathepsin L and cathepsin S in peptide generation for MHC class II presentation. J. Immunol..

[65] Davies C.J., Eldridge J.A., Fisher P.J., Schlafer D.H. (2006). Evidence for expression of both classical and non-classical major histocompatibility complex class I genes in bovine trophoblast cells. Am. J. Reprod. Immunol..

[66] Davies C.J., Fisher P.J., Schlafer D.H. (2000). Temporal and regional regulation of major histocompatibility complex class I expression at the bovine uterine/placental interface. Placenta.

[67] Streyl D., Kenngott R., Herbach N., Wanke R., Blum H., Sinowatz F., Wolf E., Zerbe H., Bauersachs S. (2012). Gene expression profiling of bovine peripartal placentomes: Detection of molecular pathways potentially involved in the release of foetal membranes. Reproduction.

[68] Zeisel S.H. (2006). Choline: Critical role during fetal development and dietary requirements in adults. Annu. Rev. Nutr..

