# Supplementary material for: Identification of Pathways Associated with Placental Adaptation to Maternal Nutrient Restriction in Sheep
Source: Genes (Basel). 2020 Sep 2;11(9):1031. doi: 10.3390/genes11091031 (PMC7565845; doi:10.3390/genes11091031)
Supplement: Supplementary file 1 [file genes-11-01031-s001.pdf]

Table S1. Primer sequences for qPCR and *in situ* hybridization analyses.

Gene	Forward/Reverse primers (5'-3')	Amplicon Size	GenBank Accession
AGR2	CCTCTCTCCTGATGGCCAGTAT<br>CAGTCAGGGATGGGTCAACAA	55	NM_001040500
B3GALT2	ACTACTAATCGCTGCGGAACCT<br>GCCCCAAGTTTGCCGAAT	64	NM_001076188
CADM1	AAGCCCCAGCCTGTGATG<br>ACGGCATGTTGAGGCATTTC	62	NM_001038558
CD37	TTCGTGGGCTTGTCCTTCAT<br>CTGAGACGGCCAGGACCTT	58	NM_001046011
CD86	GGCCGCACAAGCTTTGA<br>TTTGATTTGAACGTTGTGGAGTCT	60	NM_001038017
CTSS	TGGGAGCCCTGGAAGCA<br>TGCACTCAGAGACACCAGCTTT	59	NM_001033615
CTSS	CCTGGAAGCACAAGTGAAGC<br>GAATGGCTCGCGTCTATACC	330	NM_001033615
CXCL10	CCGTGGACTTCGGTTTTCCTA<br>GCAGGAGTAGTAGCAGCTGATATGG	66	NM_001046551
CXCL14	CCGCTACAGCGACGTGAA<br>CCTCGCAGTGCGGGTACTT	56	NM_001034410
DPYD	TGCTCCAGGTATGCAGTGCTAT<br>TTTGAGGCCAGTGCAGTAGTCTT	71	NM_174041
GATM	CCGAAGCGCTGCACTACA<br>CACCCATCCCGTTACAGTTCTT	56	NM_001045878
HDAC11	CACGGCCCGCATCATC<br>ATGAGCCCCAGGCTGTACAG	54	NM_001102056
IL12RB2	ATGGTGGGCGTTCTCTCAAT<br>GAGGGCCAAAAGGAGAACAAA	63	NM_174645
IL12RB2	TGTGCAGGAATACGTGGTGG<br>CAACGCATTGAGAGAACGCC	585	NM_174645
LIPG	GAGGGACGGCTGCAAGGT<br>TGGCAGCAGTCGGTACCA	55	XM_002697766
NUP210	CAGTGATGTTTTTTATTTGTGTCAGTTC<br>ATTTGCTCCAATCATTTCCCAGTA	92	NM_001191461
SLC44A4	GACCCCATGGAGCAAGTGA<br>GCCCTGGAAGACGCACAT	57	NM_001083442
SLCO1C1	GTGCCTGGTGGCTTGGTTA<br>GGCACAGCTGCCAAAAGAGT	58	NM_001191509
SPP1	TTCTGCCTCTTGGGCATTG<br>CTGCCAGAACTGGTCGGTTT	56	NM_001009224
STC1	TGTGATCCGGCCTGCTATG<br>ACTGATGAACGGTGACAAGTCAA	61	NM_176669
STC1	TGATCAGTGCTTCTGCAACC<br>TCACAGTCCAGTAGGCTTCG	478	NM_176669
SULF2	CCCACCACCGCCTGAA<br>GGATGATGTTGGGACGAATGT	63	NM_001192938
TUBA	GGTCTTCAAGGCTTCTTGGT<br>CATAACGACAGAGAGGCGT	54	AF251146
